# Practical independent research projects in science: a synthesis and evaluation of the evidence of impact on high school students

**DOI:** 10.1080/09500693.2018.1511936

**Published:** 2018-09-01

**Authors:** Judith Bennett, Lynda Dunlop, Kerry J. Knox, Michael J. Reiss, Rebecca Torrance Jenkins

**Affiliations:** a Department of Education, University of York, York, UK; b UCL Institute of Education, London, UK

**Keywords:** Practical work, research projects, high school, systematic review, research synthesis

## Abstract

Practical independent research projects (IRPs) are a feature of school science in a number of countries. To assess the impact of IRPs on students, a systematic review of the literature was undertaken. Thirty-nine papers met the review inclusion criteria, reporting on work from twelve countries. The review indicates that IRPs are often associated with wider initiatives such as authentic science, problem-based learning, and project-based learning. There is considerable variability in the nature of IRP work in relation to focus, models of provision, assessment, the involvement of external partners such as universities and employers, and funding, and this diversity affects judgements on the quality of the evidence base on impact. The majority of the research reviewed explored areas such as conceptual understanding, motivation to study science once it is no longer compulsory and attitudes to science, and the development of practical skills. Benefits were identified in relation to the learning of science ideas, affective responses to science, views of pursuing careers involving science, and development of a range of skills. Studies focusing on traditionally under-represented groups indicated that such students felt more positive about science as a result of undertaking IRPs. The review findings indicate that further work is needed to enhance the quality of the available evidence, to consider the ways in which IRPs can be validly assessed, to explore more fully the potential benefits for traditionally under-represented groups, and to explore more fully the potential longer-term benefits of participation in IRPs at high school level.

## Introduction and context

This paper presents the findings of a systematic review of the nature and impact of practical independent research projects (IRPs) in high school science, covering their chief characteristics, organisation and assessment, and impact on high school students’ learning of science and affective responses to science.

Practical work in school science can be very diverse: at one end of the spectrum is the ‘recipe’ approach, where a defined list of procedures is followed, while at the other is what can be termed an *extended investigation* or a *practical independent research project* (IRP). Such projects can take a wide variety of forms, but share several common characteristics. In essence, they are student-led, open-ended research investigations, often supported by a teacher and/or a university-based or industry-based researcher. Students have considerable control in respect of the question(s) they hope the practical work will answer and the way in which the work is undertaken.

Typically, though not exclusively, IRPs are undertaken by high school students, and, at the upper high school level, the outcome of the investigation is open in that neither the student nor their teacher knows exactly what the investigation will yield. Beyond this, IRPs may involve external sponsorship, and be associated with science competitions, fairs and award schemes. IRPs can also involve a diversity of assessment techniques, including the production of reports and student presentations. Frequently, they take place outside the formal school science curriculum.

For the purposes of this review, IRPs were taken to be student-led, extended open-ended investigations involving practical work, using Millar’s (2004) definition of practical work, i.e. work that encompasses activities involving students in observing or manipulating the objects and materials they are studying.

There appear to be a number of possible reasons for promoting the use of IRPs in school science lessons. First, the notion of ‘the students as scientist’ is attractive, allowing students to find things out for themselves by pursuing an idea about which they are curious. Second, IRPs are seen as a means of providing students with a realistic taste of scientific research that may motivate them to undertake further study of science. Third, the characteristics of IRPs may be identified in broader, international initiatives of the last twenty years or so, for example ‘inquiry-based science’, ‘problem-based learning’ in science and ‘authentic science’. These approaches have in common the desire to encourage students to engage in activities where at times they behave like scientists, i.e. their work is authentic in that it follows the approaches scientists take when they are trying to solve problems to which there may as yet be no agreed solution. These approaches are primarily aimed at improving cognitive and procedural outcomes for students, though also aspire to have affective benefits.

Roth (1995) argued that for school science activities to be authentic, students need to experience scientific inquiry that has features in common with scientists’ activities in that students

(1) learn in contexts constituted in part by ill-defined problems; (2) experience uncertainties and ambiguities and the social nature of scientific work and knowledge; (3) learning is predicated on, and driven by, their current knowledge state; (4) experience themselves as parts of communities of inquiry in which knowledge, practices, resources and discourse are shared; (5) in these communities, members can draw on the expertise of more knowledgeable others whether they are peers, advisors or teachers. (p1).

There are similarities between the features of authentic learning described by Roth and the characteristics associated with problem-based learning (PBL). A PBL approach involves students learning through focusing on the investigation, explanation, and resolution of meaningful problems. PBL has its origins in teaching in medical schools, but has now been used in a variety of subjects, including science. Students work collaboratively in small groups, with the teacher acting as a facilitator to guide student learning through the process of solving the problem presented (see, for example, Hmelo-Silver, 2004; Krajcik & Blumenfeld, 2006).

Inquiry-based learning is another related approach that is widely used in the context of science teaching. In a research synthesis of inquiry-based learning in science, Minner, Levy, and Century (2010) identified that one of the ways in which the term is used is to describe a pedagogical approach that teachers employ for designing or using curricula that allow for extended investigations. Drawing on the work of the National Research Council in the USA, Minner et al. (2010) cite the following as the core components of inquiry-based learning for learners: (1) they are engaged by scientifically-oriented questions; (2) they give priority to evidence, which allows them to develop and evaluate explanations that address scientifically-oriented questions; (3) they formulate explanations from evidence to address scientifically-oriented questions; (4) they evaluate their explanations in light of alternative explanations, particularly those reflecting scientific understanding; (5) they communicate and justify their proposed explanations; (6) they design and conduct investigations.

Authentic learning, problem-based learning and inquiry-based learning all emphasise open-ended investigative work, including the sort of work that occurs in IRPs, and hence have been considered in this review.

## The aims of the review

The review addressed the following questions:

1. What are the chief characteristics of practical independent research projects (IRPs), including organisation and assessment?
2. What is the quality of the research evidence base on the impact of IRPs?
3. What are the impacts of IRPs on the learning of science and affective responses to science in secondary school students’?

The impetus for the review arose from recent changes in the policy regarding the teaching and assessment of practical work as part of the national examinations for students aged 16 and 18 in England. One outcome of these changes has been a decisive move away from IRPs as part of the core curriculum which, in turn, has led to a concern that there will be a reduction in some of the learning potentially associated with IRPs. While a number of studies have been conducted into the impact of IRPs, to the best of the authors’ knowledge, a review of the associated literature has not been published. Thus the review seeks to bring together recent research into the impact of IRPs, in order to synthesise the existing literature and highlight areas suitable for further investigation.

## Review methods

The review took the form of a systematic review. Systematic reviews were introduced as a tool in educational research in the early 2000s to synthesise research findings from a range of related studies (see, for example, Gough & Elbourne, 2002; Gough, Oliver, & Thomas, 2012).

The review comprised five main stages:

1. Identification of the literature
2. Extraction of the key information from the literature
3. Production of an overview of the key features of IRP provision
4. Assessment of the quality of the available evidence in the literature on the impacts of IRPs
5. Synthesis of the evidence.

### Identification of the literature

The relevant literature was identified through a search was undertaken of the following electronic databases: the British Education Index (BEI), the Education Resources Information Centre (ERIC), PsychINFO and the Social Science Citation Index (SSCI). Additionally, 27 key informants working in the area of IRPs in a range of countries were contacted to identify potentially relevant publications. This was done to add to the robustness of the evidence base, as preliminary electronic searches indicated that there were likely to be a number of publications in the form of grey literature (e.g. reports commissioned by IRP providers) that might not otherwise be identified.

In practice, the identification of the relevant literature posed one of the major challenges for the review as IRP activity can be encompassed by a number of other activities. These include: authentic science, independent and/or extended practical work, inquiry-based science, investigative work, practical work, problem-based science and project work. IRP work is also often associated with science competitions and fairs. This diversity in terms necessitated particularly extensive literature searches in order to include all the above terms. Full details may be found in the technical report (Bennett, Dunlop, Knox, Reiss, & Torrance Jenkins, 2016).

The electronic searches identified 1,403 publications, with a further eleven included based on information from the key informants. To identify the literature that focused specifically on the review questions, the following inclusion criteria were developed:

1. One or more of the review questions addressed
2. Focus on students in 11–19 age range
3. Focus on science subjects
4. Date of publication after 2000
5. Students had major input into the question(s) addressed by the IRP
6. Students had major input into the design of the IRP
7. Included practical work
8. Required more than 10 hours of work
9. Entailed production of a report or comparable output
10. Data gathered systematically on students’ learning of science and/or affective responses to science
11. Some form of assessment or accreditation
12. Publication written in English.

Criteria 3 and 5–7 were set as they encompassed the definition of IRPs used in this review. The review was limited to work undertaken with students aged 11–19 as this covers the high school period where the majority of IRP work takes place in schools. The search covered the period from 2000 onwards in order to focus on recent work. The criterion of an extended period of time for the IRP work was set to avoid the inclusion of short, even single-lesson, investigations which have become common in some countries. The requirement to produce an output of some form was set to yield information on how IRPs might be assessed. When the inclusion criteria were applied, a total of 39 publications resulted.

## Extracting key information from the literature

In addition to the wide range of search terms required and the extensive resulting literature, several other factors contributed to the complexity of the review. A number of different research approaches and data collection techniques were used across the studies as a whole, and this research varied in rigour and detail reported. The studies included in the review took place in a range of contexts within and beyond school settings, and in a range of science disciplines. Thus, a particular challenge for extracting key information from papers was the development of a bespoke data extraction instrument to record systematically the wide range of features and considerable variation in practice associated with IRP work and research into its impact. A pilot version was developed by two of the researchers and independently tested on a subset of papers. Minor modifications were made as a result of this to ensure the instrument captured all the essential information. The resulting bespoke data extraction instrument focused on the following information:

- Background information (author(s), year of publication, title, source, country of origin, author details)
- The aims and research questions of the study
- The name of the associated IRP scheme (if applicable) and a short account of the IRP, including: its aims; principal characteristics (e.g. optional or compulsory, duration, organisational details, degree of student control over questions, whether undertaken by individuals or in teams, input from teacher or others, e.g. intern, university researcher); assessment/accreditation arrangements; associated funding
- Design of the study and sample details
- Data collection techniques (including checks for the reliability and validity of instruments)
- Methods of data analysis (including assessment of reliability and validity)
- Findings (including any impacts on students’ learning, affective responses/attitudes and subject choices or career intentions).

## Findings: characteristics of IRPs and overview of provision

The overview is based on 39 publications that report data on impacts of IRPs in enough detail to understand the nature of the IRP and any effects. The publications covered IRP activity in twelve countries, as shown in Table 1.

**Table 1.** Country of study.

Country	Publications
Australia	2
Ireland	1
Israel	1
Netherlands	1
New Zealand	1
Qatar	1
Singapore	1
Spain	1
Taiwan	1
Turkey	2
UK	8
USA	19
More than one country	2
TOTAL	39

The majority were from the USA (17 studies) and the UK (8 studies), with two studies coming from each of Australia and Turkey, and single studies from Ireland, Israel, The Netherlands, New Zealand, Qatar, Singapore, Spain and Taiwan.

Table 2 summarises five contrasting IRP models to illustrate the diversity in student work undertaken for IRPs and the outcomes reported.

**Table 2.** Details of five of the IRPs.

Publication	Burgin et al. (2012)	Charney et al. (2007)	Chin and Chia (2004)	Grant (2007)	Hubber et al. (2010)
Source	*Research in Science Education*	*International Journal of Science Education*	*Journal of Biological Education*	http://www.britishscienceassociation.org/crest-evaluation	*Teaching Science*
Name of IRP	Student Science Training Programme (SSTP)	Waksman Student Scholars Programme (WSSP)	No specific name	CREST (CREativity in Science and Technology	BHP Billiton Science Awards (Commonwealth programme linked to CREST awards)
Country where IRP carried out	USA (Florida)	USA (New Jersey)	Singapore	UK	Australia
Student age (years)	16–17	15–17	14–15	11–19	11–15
Subject area	Chemistry	Biology (genetics)	Biology (food and nutrition)	Science	Science
External groups involved	University (mentor scientists)	University (mentor scientist)	None	Some projects involve employers and universities	‘External professionals’ mentioned
Nature of student participation	Individuals	Teams	Teams	Teams	Individuals
When undertaken	Summer residential school (seven weeks)	Summer school (four weeks) plus 25 hours in-school follow-up	18 weeks during school time	During and outside school time over several weeks	During and outside school time over several weeks
Linked events	None	None	None	Locally organised events	Can be presented at science fairs
External funding	Charitable grant	National Institutes of Health, the National Science Foundation, industrial funding and funding from partner university	None	Charitable and government grants	BHP Billiton (industrial sponsor)
Number of participating students	18 (including seven females and seven from ethnic minority groups)	30 (including 17 females and 18 from ethnic minority groups);	39	512 students 62 teachers	65
Student product	Research report and presentation	Poster presentation	Team report and presentation	Report and presentation	Not explicitly stated
Impact measures / data collection approaches	Student interviews Mentor (scientist) interviews Concept maps prepared by students	Student diaries Assessment of conceptual knowledge Assessment of views of nature of science	Student questionnaire Student interviews Observation, audio and videotapes of group work	Student questionnaire Teacher questionnaire Student focus groups Teacher focus groups	Student questionnaire Teacher interviews Student interviews State organiser interviews
Links to wider initiatives	Authentic science	Authentic science	Problem-based learning	None stated	Authentic science
IRP focus	Projects on ‘genuine unanswered questions’ in chemistry	Open-ended projects linked to genetics research	Projects based on newspaper reports of food and nutrition issues	A variety of open-ended science projects	A variety of open-ended science projects
Reported outcomes	Students reported improved scientific knowledge; this was supported by data from concept maps Four students reported increased interest in pursuing a career in research science	Improved student knowledge, broader awareness of nature of science, promotion of collaborative learning environment	Most students viewed IRP work positively and enjoyed freedom to work in new ways Students not very confident about making presentations PBL approaches are time-consuming	78% of students rated CREST as good or very good 16% reported increased likelihood of undertaking further study in STEM subjects Teachers reported improved post-compulsory uptake of science subjects	Most students reported increased interest in science Anecdotal data from teachers indicated increased post-compulsory uptake of sciences Teachers felt IRP provided a positive experience for students normally less successful in science

There were three principal contexts in which students engaged in IRPs. In some cases, undertaking IRPs was linked to national policies/agendas. For instance, several USA studies reported on interventions that had secured funding for local initiatives through linking them to policy statements by organisations such as the AAAS (American Association for the Advancement of Science) or the NAS (National Academy of Sciences) (Adams et al., 2009; Dolan, Lally, Brooks, & Tax, 2008; Gibson & Chase, 2002; Sahin, 2013). Secondly, as noted earlier, IRPs were very often associated with wider initiatives, including: authentic science, for instance in Israel (Zion et al., 2004), The Netherlands (Bulte, Westbroek, de Jong, & Pilot, 2006) and the USA (Burgin, Sadler, & Koroly, 2012; Dolan et al., 2008; Rivera Maulucci, Brown, Grey, & Sullivan, 2014); problem-based learning, for instance in Qatar (Faris, 2008) and Singapore (Chin & Chia, 2004); and project-based learning, for instance in the USA (Krajcik & Blumenfeld, 2006; Schneider, Blenis, Marx, & Soloway, 2002).

Thirdly, a number of IRP activities were linked to non-governmental groups with a specific interest in promoting IRPs as a way of providing young people with authentic experiences of working as a scientist. Such initiatives typically involved school-university partnerships and included the CREST awards which are run in several countries, including the UK and Australia (British Science Association, 2014; Grant, 2007; Moote, Williams, & Sproule, 2013) and, in the UK, the Nuffield Research Placements scheme (Nuffield Foundation, 2013), The Royal Society Partnerships Grants scheme (Jenkins & Jeavans, 2015) and the Authentic Biology Project funded by the Wellcome Trust (Colthurst et al., 2015; Finegold, 2015).

Just over half of the IRPs (20) involved people outside schools. The largest group of these groups comprised university science staff or students, acting as advisers/mentors. Examples in the USA include O’Neill and Polman (2004), Burgin et al. (2012), Charney et al. (2007), Campbell and Neilson (2009) and Schneider *et al*. (2013). Other examples include Symington and Tytler (2011) in Australia, Diaz-de-Mera et al. (2011) in Spain, and one IRP programme taking place across six European countries (Dijkstra & Goedhart, 2011). Around a quarter of the IRPs included industrial partners and employers, e.g. Welch (2010) and Duran, Höft, Lawson, Medjahed, and Orady (2014). Less frequently, local voluntary groups and parents were involved, e.g. Adams et al. (2009).

A small number of publications reported on IRPs undertaken by groups of schools or individual teachers in their own school and not involving any partners: Chin and Chia (2004), Zion et al. (2004), Chien and Karlich (2007), Haigh (2007), Faris (2008), and Balmer (2014).

IRPs were most prevalent at upper high school level, i.e. for ages 16–19 (17 studies), as shown in Table 3.

**Table 3.** Student age range.

Age of students	Publications
Lower high school (age 11–14)	7
Middle high school (age 14–16)	6
Senior high school (age 16–18)	11
High school (ages 11–16)	1
High school (ages 11–19)	7
High school (age not specified)	7
TOTAL	39

Of the 16 studies focusing on one of the science disciplines, rather than simply being ‘science’, biology IRPs (7) were more common than chemistry (2) or physics (2). Even within a specific science discipline, there was considerable diversity in the topic focus. Biology-related IRPs, for example, explored diet, food and nutrition (Chin & Chia, 2004; Faris, 2008), genetics (Charney et al., 2007), plant biology (Dolan et al., 2008), environmental science (Faris, 2008), pharmacology (Sikes & Schwartz-Bloom, 2009), the carbon cycle (Dijkstra & Goedhart, 2011), and biomedical science (Colthurst et al., 2015; Finegold, 2015).

Two models predominated for the creation of time for IRPs. Most commonly, they were undertaken during normal school hours, sometimes supplemented with time in after-school clubs (e.g. Brand, Collver, & Kasarda, 2008; Hong, Chen, & Hwang, 2013; Sahin, 2013). Typically, such IRPs were of six weeks to a year’s duration (e.g. Chin & Chia, 2004; Dijkstra and Goedhart, 2011; Faris, 2008; Hong *et al*., 2013; O’Neill & Polman, 2004). Occasionally, time was created within schools through ‘intensive pull-outs’ whereby students were taken off their normal timetable for a period to be dedicated to IRP work (e.g. Rivera Maulucci et al., 2014). Five of the IRPs were associated with dedicated out-of-school events such as one- or two-week summer schools and camps (e.g. Akinoglu, 2008; Burgin et al., 2012; Gibson & Chase, 2002; Metin & Leblebicioglu, 2011). In two cases (Brand et al., 2008; Yasar & Baker, 2003), the IRPs were linked to participation in science competitions or fairs.

There was only one country, Ireland, where the IRP work was a compulsory component of a national end-of-course science examination (Kennedy, 2014). In England, the IRPs could, for students aged 16 or over, optionally be entered for a national qualification (Daly & Pinot de Moira, 2010).

In just under one third of the cases (12), participation in the IRP was compulsory. In around half the IRPs (20), students participated as part of a team, with around a quarter (10) requiring individual participation. In a small number of instances, students could choose between team or individual participation.

The majority of the IRPs required the generation of one or more products, as shown in Table 4. Written reports (19) and presentations (17) predominated, with many any IRPs requiring both. Occasionally, students were asked to produce a physical artefact or write a reflective diary.

**Table 4.** Student products (39 studies, some had more than one product).

Student products	Publications
Written report	19
Presentation	17
Artefact	1
Student reflective diary	2
Report for external examination	1
No product required (explicit statement)	1
Not specified	14
TOTAL	54

Fifteen of the IRP programmes were supported by external funding. Characteristically, this was associated with work involving partnerships with universities, employers or other groups. Where work was required for external examination or specific to one school, it was unfunded. Typically, funding for IRPs came from grants secured from national funding organisations such as government, research councils and charitable bodies with an interest in science education, or from industrial sponsors. Examples included funding from BHP Billiton, a global mining company based in Australia (Symington & Tytler, 2011), the Cosmos Foundation in Texas (Sahin, 2013), the US NSF (National Science Foundation) (O’Neill & Polman, 2004) and the Scientific and Technological Research Council of Turkey (Metin & Leblebicioglu, 2011).

Most of the funding supported regional or local initiatives, though there were examples of national initiatives including, in Australia, the BHP Billiton funding, and, in the UK, the CREST awards^1^, the Nuffield Partnerships scheme^2^ and the Royal Society’s Partnership Grants scheme^3^.

Some studies reported data on traditionally under-represented groups in science, focusing on gender, ethnicity and socio-economic status, for example, in the USA, Yasar and Baker (2003), Sikes and Schwartz-Bloom (2009) and Sonnert, Michaels, and Sadler (2013), and in the UK, the Nuffield Foundation (2013) and the British Science Association (2014). In two cases in the USA (Duran et al., 2014; Rivera Maulucci et al., 2014), the IRP work formed part of a programme intentionally developed for students with backgrounds typically under-represented in science.

## Findings: quality of evidence

In evaluating the quality of the evidence, some notes of caution need to be sounded in relation to factors that could result in bias in the evidence base. The review revealed that research into impact of IRPs was most often conducted by those associated with the funding agencies and with the development and/or running of the IRP, risking confirmation bias in the findings. Very few of the studies in this review had commissioned external evaluations, with Grant (2007) and Jenkins and Jeavans (2015) being exceptions.

Another source of potential bias concerns the nature of the data collected. Frequent use is made of data provided by the people involved in IRP work. Many of the adults who can provide data (teachers, employers, and university-based scientists) are already likely to be very sympathetic to the aims of IRPs.

A challenge in synthesising the evidence arises from the diversity in provision and execution of IRPs, which is reflected in a corresponding diversity in the aims and range of measures used to assess impact.

The main sources of data were students and their teachers, with the most of the work focusing on the impacts on students. Impacts on understanding of concepts, practical skills, cross-disciplinary skills (e.g. working collaboratively in teams), attitudes towards science and motivation to continue with science after it was no longer compulsory were explored. There was also a cluster of studies focusing on impacts on traditionally under-represented groups in relation to gender, socio-economic status and ethnic background. Studies that focused on teachers and others involved (e.g. IRP providers, university scientists/mentors, employers, state/regional organisers) explored views of the impact of IRPs on students together with views on the potential benefits and drawbacks of their own participation in IRPs.

Table 5 summarises the principal foci of research into the impact of IRPs in cognitive and affective dimensions.

**Table 5.** Focus of study.

Focus	Examples of studies including this focus	Country
Students’ conceptual understanding	Burgin et al. (2012)	USA
	Krajcik and Blumenfeld (2006)	USA
	Sahin (2013)	USA
	Schneider et al. (2002)	USA
Students’ views of the nature of science	Metin and Leblebicioglu (2011)	Turkey
Development of students’ scientific literacy	O’Neill and Polman (2004)	USA, Canada
Development of students practical and experimental skills	Chien and Karlich (2007)	USA
	Grant (2007)	UK
	Yasar and Baker (2003)	USA
	Zion et al. (2004)	Israel
Development of students’ use of technology	Duran et al. (2014)	USA
Development of students’ more general skills, such as collaborative/team working	Charney et al. (2007)	USA
	Faris (2008)	Qatar
	Grant (2007)	UK
Students’ attitudes to science	Faris (2008)	Qatar
	Gibson and Chase (2002)	USA
	Welch (2010)	USA
	Yasar and Baker (2003)	USA
Students’ creativity	Haigh (2007)	New Zealand
	Hong et al. (2013)	Taiwan
Student motivation	Moote et al. (2013)	UK
Student self-efficacy	Sikes and Schwartz-Bloom (2009)	USA
More general student responses to IRPs	Diaz-de-Mera et al. (2011)	Spain
	Finegold (2015)	UK
Barriers to student participation	Nuffield Foundation (2013)	UK
Teachers’ view of IRPs	Finegold (2015)	UK
	Chin and Chia (2004)	Singapore
	Kennedy (2014)	Ireland
Views of other people (e.g. science mentors, employers) about their participation in IRPs	Symington and Tytler (2011)	Australia
Exploration of effects of participation in IRPs of traditionally under-represented groups	Duran et al. (2014)	USA
	Rivera Maulucci et al. (2014)	USA
	Sikes and Schwartz-Bloom (2009)	USA
	Sonnert et al. (2013)	USA
	Yasar and Baker (2003)	USA

None of the studies employed randomised controlled trials; nine adopted some form of experimental design which involved making comparison between participants and non-participants in IRP activities (Finegold, 2015; Gibson & Chase, 2002; Jenkins & Jeavans, 2015; Krajcik & Blumenfeld, 2006; Moote et al., 2013; Sahin, 2013; Schneider et al., 2002; Welch, 2010; Yasar & Baker, 2003).

The predominant techniques used for gathering data from students were questionnaires, tests of understanding, inventories on affective aspects, and interviews and focus groups to explore students’ views of IRPs. Occasionally, data were drawn from student presentations, student reports on their IRP work, student reflective diaries, observations of students undertaking IRP work, and datasets such as external test and examination results. Where quantitative data were gathered, it was very rare for the reports of studies to report details of any checks on reliability and validity with research instruments or data analysis.

Table 6 summarises the impact outcome measures and the nature of the data gathered in the studies.

**Table 6.** Impact outcome measures: examples of data collected.

Data	Examples of studies collecting such data	Country
*From students*		
Measures of conceptual understanding	*Charney et al. (2007)	USA
	*Sikes and Schwartz-Bloom (2009)	USA
Measures of views of nature of science	*Charney et al. (2007)	USA
Practical abilities	*Yasser and Baker, (2003)	USA
Attitude inventory	*Krajcik and Blumenfeld (2006)	USA
	*Grant (2007)	UK
Motivation inventory	*Moote et al. (2013)	UK
Self-efficacy inventory	*Sikes and Schwartz-Bloom (2009)	USA
Student self-report data (questionnaires, interviews, focus groups, diaries)	Akinoglu (2008)	Turkey
	*Bulte et al. (2006)	The Netherlands
	Daly and Pinot de Moira (2010)	UK
	*Gibson and Chase (2002)	USA
	*Grant (2007)	UK
	*Haigh (2007)	New Zealand
	*Jenkins and Jeavans (2015)	UK
	*Nuffield Foundation (2013)	UK
	*Sikes and Schwartz-Bloom (2009)	USA
	Sonnert et al. (2013)	USA
Student presentations	*Faris (2008)	Qatar
	*Sikes and Schwartz-Bloom (2009)	USA
*From teachers*		
Teacher self-report data (questionnaires, interviews, focus groups, diaries)	*Grant (2007)	UK
	*Jenkins and Jeavans (2015)	UK
	Kennedy (2014)	Ireland
	*Rivera Maulucci et al. (2014)	USA
*From other people*		
Researcher involved in IRP self-report data (questionnaires, interviews)	*Jenkins and Jeavans (2015)	UK
	*Nuffield Foundation (2015)	UK
Interview with others (IRP providers, IRP regional/state organisers, employers, parents, key informants)	*Grant (2007)	UK
	*Hubber et al. (2010)	Australia
	*Jenkins and Jeavans (2015)	UK
	*Symington and Tytler (2011)	Australia
*Other data sources*		
Assessment of student report on IRP	*Bulte et al. (2006)	The Netherlands
External examination result	Kennedy (2014)	Ireland
Observation of IRP activity	*Bulte et al. (2006)	The Netherlands
Document study	*Nuffield Research Placements, (2013)	UK
Use of external datasets	*Krajcik and Blumenfeld (2006)	USA
	Sahin (2013)	USA

* = more than one data source gathered in study.

The wide variety of outcome measures points to one of the most prominent features of research into the impact of IRPs, which is the very disparate approach to judging the impact of IRPs.

Studies gathering data on cognitive impacts include those of Krajcik and Blumenfeld (2006), Burgin et al. (2012) and Sahin (2013) in the USA. Studies gathering data on the impact of IRPs on students’ attitudes to science include Faris (2008) in Qatar, and Gibson and Chase (2002), Yasar and Baker (2003) and Welch (2010) in the USA. Other studies on affective responses include students’ motivation (Moote et al., 2013), and students’ self-efficacy (Sikes & Schwartz-Bloom, 2009). Other aspects explored include views of the nature of science (Metin & Leblebicioglu, 2011), development of students’ practical and experimental skills (Chien & Karlich, 2007; Grant, 2007; Yasar & Baker, 2003; Zion et al., 2004), and development of more general skills in students, most often related to as collaborative working in teams (Charney et al., 2007; Faris, 2008; Grant, 2007).

Studies contained varying amounts of detail on the techniques employed to gather data on the impact of IRPs. As might be anticipated, full reports contained more detail than journal papers, particularly on instrument design. There were no examples of replication studies and virtually all the studies gathered data using specifically-designed instruments, though a few used state or national test instruments of subject knowledge (Daly & Pinot de Moira, 2010; Krajcik & Blumenfeld, 2006; Schneider et al., 2002) or existing, validated instruments to measure student characteristics such as motivation (Moote et al., 2013). Most studies drew on at least two sources of data.

In order to evaluate the quality of the evidence base as a whole, the criteria widely employed in making such judgements about systematic reviews were used (see, for example, Gough et al., 2012). These take into account the declared aims of the studies, the hypotheses and research questions, strategies employed for identifying the sample, the nature and extent of the data gathered, the appropriateness of how the data were collected and the methods employed to analyse the data (including information on reliability and validity checks) and the extent to which the conclusions appear sound in relation to the data gathered.

With the exception of an over-reliance on self-reported data in some cases, the studies included in the review appeared to have adequate or good designs, with no obvious adverse effects arising from researcher involvement in the design and undertaking of studies. However, the quality of individual studies is offset by the diversity in focus and in the instruments used, with one of the most prominent features of the work as a whole being the comparatively uncoordinated and unsystematic approach to gathering evidence on, and judging the impact of, IRPs. This diversity poses a challenge for the synthesis of data, and the current evidence base would not permit a meta-analysis of data (i.e. an analysis that quantitatively aggregates data across studies, as is common in medical studies, leading to an overall conclusion as to the effects of IRPs with effect sizes or odds ratios).

## Findings: the evidence on impact

A wide range of potential impacts of IRPs are reported, particularly on students, with studies reporting on students’ responses to undertaking IRPs, effects on students’ learning and effects on students’ attitudes to science, including attitudes towards pursuing a career in science. Within this, some studies explore elements to do with widening participation issues. Some studies also report on impacts on teachers and, less frequently, impacts on other participating adults such as university scientists and employers.

Gains in students’ learning are reported (e.g. Burgin et al., 2012; Daly & Pinot de Moira, 2010; Krajcik & Blumenfeld, 2006; Rivera Maulucci et al., 2014; Sahin, 2013). Whilst most studies take their data from instruments devised specifically for the study being reported, three studies report on gains in students’ learning based on data from external state or national instruments (Daly & Pinot de Moira, 2010; Krajcik & Blumenfeld, 2006; Schneider et al., 2002).

Improvements in students’ attitudes to science and motivation in science are also reported (British Science Association, 2014; Faris, 2008; Gibson & Chase, 2002; Hubber, Darby, & Tytler, 2010; Jenkins & Jeavans, 2015; Krajcik & Blumenfeld, 2006; Moote et al., 2013; Welch, 2010; Yasar & Baker, 2003). As with student learning, most studies draw on instruments designed specifically for the study, with the exception of Moote et al. (2013), where an existing instrument for motivation was used.

All the studies exploring the impact of IRPs on students’ interest in pursuing careers in science/science-related areas report increased numbers of students indicating that their participation in IRPs meant they were more likely to consider careers in science (e.g. Adams et al., 2009; Hubber et al., 2010; Jenkins & Jeavans, 2015). Students indicated that this was primarily due to increased awareness of the range of careers and the varied nature of work in which people with science qualifications engage. Improved practical skills and abilities are also reported (e.g. Adams, 2009 in the USA; British Science Association, 2014 in the UK).

One finding of particular interest to emerge from some studies in the USA and the UK was potential benefits to traditionally under-represented groups in science in relation to ethnicity and socio-economic status. In the USA, four studies report improved engagement for such students (Duran et al., 2014; Rivera Maulucci et al., 2014; Sonnert et al., 2013; Yasar & Baker, 2003), with Sikes and Schwartz-Bloom (2009) noting interest declining slightly. In the UK, the British Science Association (2014) found that uptake of IRPs was higher than average for students from lower socio-economic groups. The Nuffield Foundation (2013) reports particular benefits in engagement for students from disadvantaged backgrounds.

Where negative notes about involvement in IRPs were sounded in the studies, the focus was often on practical matters. These included teachers reporting that IRPs were unduly time-consuming (Faris, 2008), had a negative effect on the time available for teaching other aspects of science (Kennedy, 2014), or adversely affected the school’s ability to deal with external inspections (British Science Association, 2014; Jenkins & Jeavans, 2015). Additionally, some teachers reported lacking the confidence to run IRPs (British Science Association, 2014; Jenkins & Jeavans, 2015) and problems with the identification of external partners (Jenkins & Jeavans, 2015). One study (Kennedy, 2014) reported that teachers felt IRPs discouraged students from considering further study of science subjects.

Very few details on assessment criteria for IRPs are reported in the studies, making it difficult to judge the evidence on assessment and hence measures of validity. This absence also hinders comparisons between the impact of more traditional approaches to practical work with that of IRPs, with none of the studies reporting on this aspect.

## Conclusions

IRPs are often associated with country-wide policy initiatives in science education. Generally, they are perceived as valuable and important by a range of groups with interests or involvement in science education. Such groups include teachers, scientific researchers, industrial employers, charitable foundations, professional societies and learned bodies. The notion of allowing students to find out what it is like to be a scientist is seen as particularly attractive. In addition to the benefits reported for students, benefits are also claimed for other people involved, including the links built between students, teachers, schools and employers. However, the review shows that only a minority of students in any country are normally offered the opportunity to undertake an IRP.

The review reveals a diversity in the conceptualisation and implementation of IRPs, posing challenges for synthesising the evidence and making judgments about the impact and effectiveness. The review points to more work being needed to establish that the potential benefits of IRPs are such that they should definitely be used more widely in the school curriculum. A key element of such additional work would be focusing on improving the quality of the available evidence. This could be achieved by some relatively straightforward steps. First, the rigour of studies could be enhanced by making more use of experimental or quasi-experimental study designs. As IRPs are optional in most cases, this facilitates the gathering of data from control and intervention groups. Where such designs are not feasible, there are examples in this review (e.g. Krajcik & Blumenfeld, 2006) where good use has been made of existing data sets on student performance to enable comparisons to be made between students who have undertaken IRPs and wider populations. Secondly, there is an over-reliance in some studies on self-report data. More rigour would be introduced through the gathering of more than one source of data. Thirdly, and whilst recognising that methods of evaluation will need to vary in order to accommodate the particular features of specific IRPs types and programmes, those undertaking research on impact need to make greater use of previous work, particularly in relation to focus and methods employed to gather data. Considerable benefits would be conferred through greater agreement about the areas in which to collect data: increased use of existing, validated instruments, rather than excessive development of new instruments, would facilitate the building up of a more coherent evidence base. Areas of particular importance in which to gather data are students’ learning of science concepts, students’ views of the nature of science, and students’ affective responses to participation in IRPs. In the second and third of these areas, a number of instruments already exist and could be utilised. The learning of science concepts is likely to require more work, given that many IRPs involve an in-depth study of a relatively narrow area.

While this review has focused on the impact of IRPs on students, the studies have pointed to a number of other practical factors that would need to be considered were schools to be given more encouragement to offer IRPs. IRPs place particular demands on students, teachers, universities and employers that are not associated with more standard school provision. The demands relate to resource (time and money), skills required by teachers and other adults involved in IRPs, and supporting infrastructure. External funding to support IRPs currently comes from a range of sources, including government agencies, charitable bodies, industrial sponsors and other groups that fund research. There are some examples of co-ordinating bodies being established that could play a useful role in supporting IRP work (e.g. the Institute for Research In Schools [IRIS] in the UK), through identifying funding opportunities, training opportunities, and interested external partners.

In addition to the need for improvement in the quality of the evidence base, the review findings point to three particular areas that would benefit from further research to inform any decision on making more widespread use of IRPs. The first of these is the assessment of IRPs, where there is a dearth of information in the studies in the review. As noted earlier, one of the motives for undertaking IRPs is to give students the opportunity to ‘be like a scientist’. Given this, it would be useful to involve practising scientists in discussions about what being like a scientist means in operational terms, and a consideration of what this means for assessment of IRPs. One avenue of potential utility is that of *threshold concepts* (Land, Meyer, & Smith, 2008; Meyer & Land, 2003), namely concepts that substantially change how students view their discipline and which change the learner’s approach to, and perception of, learning in their subject. In the context of IRPs, threshold concepts are those concepts that are central to being able to think and act like a research scientist, which result in students seeing science in a new light, and may alter their feelings towards science. The second area of work relates to the emerging evidence of the possible benefits of IRPs for increasing engagement with science in students from traditionally under-represented groups. Here, there would be merit in undertaking case studies of particular groups of students in order to characterise the features of IRPs that appear to have a positive impact on views of science and engagement with science. Finally, given the range of short-term potential benefits reported for IRPs, it is important to explore possible longer-term benefits through looking at the impact of IRPs on subsequent subject and career choices.

## Notes

1. http://www.britishscienceassociation.org/crest-awards.

2. http://www.nuffieldfoundation.org/nuffield-research-placements.

3. https://royalsociety.org/grants-schemes-awards/grants/partnership-grants/.
